# Characterization of the Ligamentum Mucosum in the Feline and Canine Stifle

**DOI:** 10.1111/ahe.70144

**Published:** 2026-06-19

**Authors:** Nathan Thomas Ko, Elizabeth Moran Woodward

**Affiliations:** ^1^ Department of Biomedical Sciences, School of Veterinary Medicine University of Pennsylvania Philadelphia Pennsylvania USA

**Keywords:** canine stifle, feline stifle, ligamentum mucosum

## Abstract

In humans, the ligamentum mucosum (LM) is described as a ligamentous structure originating from the femoral intercondylar notch and inserting into the infrapatellar fat pad. Proposed clinical implications include knee stabilization, contribution to post‐operative revascularization of adjacent structures, and causation of anterior knee pain if inflamed. Published reports of the LM are rare in dogs and, to our knowledge, none exist for cats. Because common veterinary gross anatomy texts omit the LM from their descriptive anatomy of the stifle, students sometimes mistake the LM for the cranial cruciate ligament. Therefore, a description of the LM in the feline and canine stifle would serve as an important veterinary anatomy learning resource. The aim of this project was to characterize the LM in the cat and dog. Stifles were dissected from 62 cat hindlimbs (*n* = 24 preserved, *n* = 38 fresh) and 47 dog hindlimbs (*n* = 9 preserved, *n* = 38 fresh). The presence or absence of the LM was determined and described. Representative samples were processed for haematoxylin and eosin staining. Grossly, the LM was found bilaterally in 95.2% of cats and in 83.0% of canine limbs, appearing as an elastic, friable band of white‐to‐pink tissue tethering the infrapatellar fat pad to the femoral intercondylar notch. Histological samples revealed collagen fibrils, vascular structures and neural tissue. These data provide evidence of the LM in the cat and dog, and bolster currently available anatomic educational resources. The presence of the LM in the canine and feline stifle merits further investigation into its function in health and disease states.

## 1 Introduction

### 1.1 Stifle Anatomy and Physiology

The stifle joint, or knee, is a complex condylar synovial joint of the femur, tibia, patella and fibula in the pelvic limb of many vertebrate species. The stifle is comprised of the femorotibial (with lateral and medial components), femoropatellar, and proximal tibiofibular articulations, and its primary actions are extension and flexion. A fibrous joint capsule surrounds, stabilizes, and lubricates the stifle, and is partitioned into three compartments which communicate with one another (Payne and Constantinescu 1993; Agnello 2022; Carpenter and Cooper 2000; Hermanson et al. 2020). Within the capsule, the fibrocartilaginous medial and lateral menisci serve as cushions between the medial and lateral femoral condyles and corresponding tibial condyles, respectively.

**FIGURE 1:**
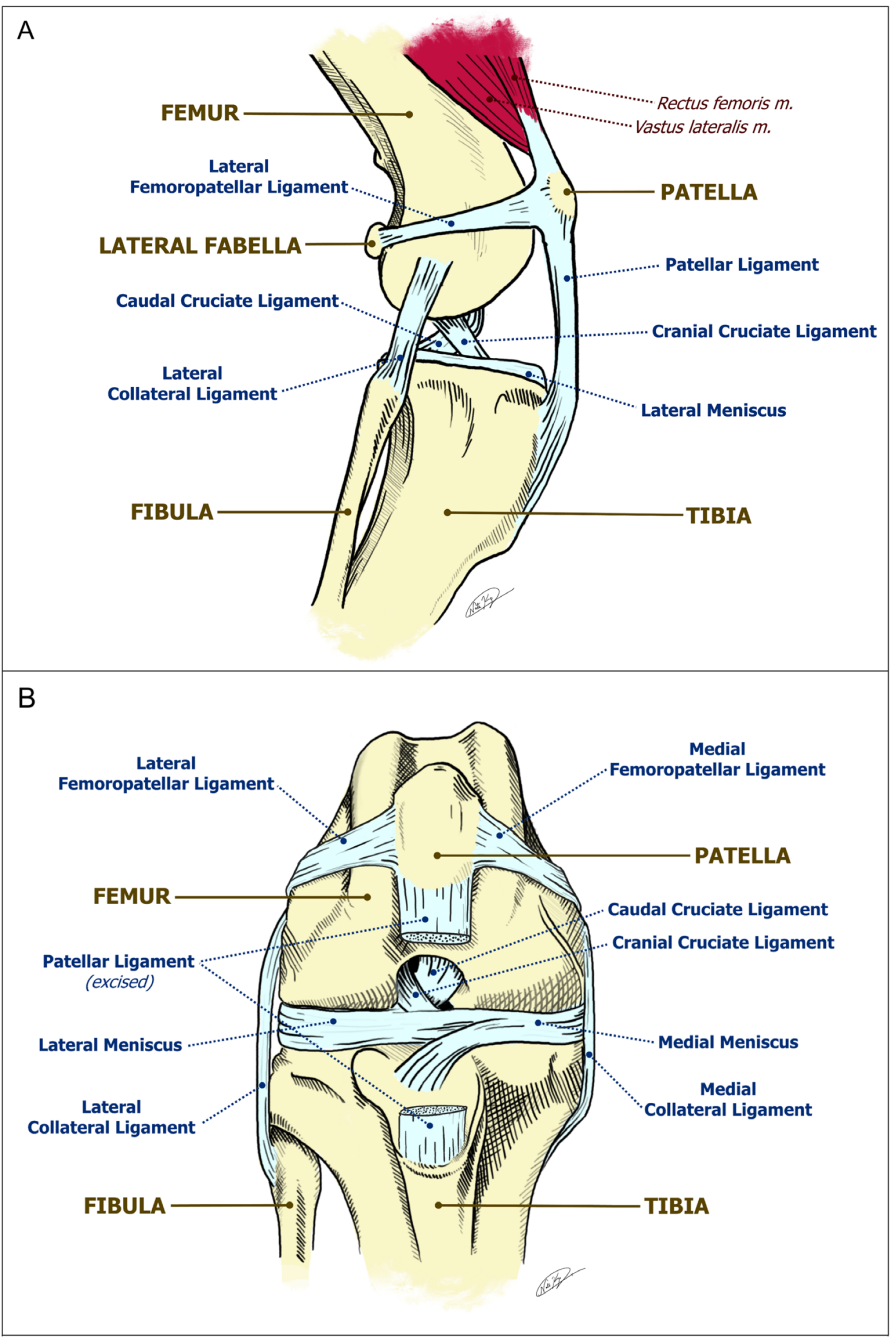
Ligaments of the right canine stifle. Level of detail is typical of the standard in most veterinary anatomical texts. (A) Cranial view. (B) Lateral view.

There are several ligaments that stabilize the stifle, and the bulk of the stabilization is provided by the medial and lateral collateral ligaments (ligamenta collaterale mediale and laterale), and the cranial and caudal cruciate ligaments (ligamentum cruciata craniale and caudale). The collateral ligaments prevent abduction, adduction, and rotation of the stifle during stifle extension, while the cruciate ligaments prevent craniocaudal sliding of the femur and tibia relative to each other. Near the cruciate ligaments is the meniscofemoral ligament (ligamentum meniscofemorale), which runs from the caudal horn of the lateral meniscus to the lateral surface of the medial femoral condyle. From the patella extend three stabilizing ligaments: the patellar ligament (ligamentum patellae) distally to the tibial tuberosity, and the two femoropatellar ligaments medially and laterally (ligamenta femoropatellare) to the fabellae, which are located on the caudal area of the joint (Payne and Constantinescu 1993; Agnello 2022; Carpenter and Cooper 2000; Hermanson et al. 2020). Finally, the patella serves as an attachment point for the quadriceps femoris, biceps femoris, and sartorius muscles, and these attachments further stabilize the stifle (Figure 1).

### 1.2 Ligamentum Mucosum in Human Medicine

In humans, synovial plicae are ligamentous structures formed when synovial membranes involute during embryonic development (Casadei and Hermena 2023). One of these, the infrapatellar plica, commonly and henceforth referred to as the ligamentum mucosum (LM), originates from the femoral intercondylar notch and inserts into the infrapatellar fat pad (corpus adiposum infrapatellare) (Singh et al. 2021). Its microanatomical composition is similar to that of surrounding ligaments of the knee, consisting of collagen (predominately Type I), vasculature, and neural tissue (Gonera, Kurtys, et al. 2023). Previously considered only a vestige of embryological development, its proposed clinical relevance now includes roles in anterior knee pain or “plica syndrome” (Casadei and Hermena 2023), and notably, joint stabilization and prevention of knee osteoarthritis (Martin et al. 2023). Arthroscopic excision of the LM has been attempted as a treatment for knee pain, popping, or snapping (Demirag et al. 2006).

### 1.3 Ligamentum Mucosum in Veterinary Medicine

In dogs and cats the LM remains sparsely described, and it is not a currently listed structure in the most recent edition of the *Nomina Anatomica Veterinaria* (International Committee on Veterinary Gross Anatomical Nomenclature [I.C.V.G.A.N.] 2017). A 1999 report describes a “ligamentum synoviale infrapatellare” in the stifles of ten dogs with attachments and composition consistent with those of the human LM (Sommer et al. 1999). The same study also explored the microanatomy of the ligament and determined that it was comprised of blood vessels, collagen, elastin, and nerves (although the nerves did not appear to be sensory in nature).

The authors of this study were unable to find any descriptions of a feline LM or homologous structure in the literature. Anecdotally, veterinary orthopaedic surgeons at Ryan Veterinary Hospital have observed a structure during arthroscopic procedures on dogs. Dubbed the “new intern cruciate ligament” for the propensity of interns and surgery residents to initially misidentify it as the cranial cruciate ligament, this structure's position within the stifle matches the known location of the human LM, but it is absent from oft‐referenced veterinary texts (Figure 1).

Because common veterinary anatomical texts omit the LM from their descriptive anatomy of the stifle, students sometimes mistake the LM for the cranial cruciate ligament when learning anatomy. Therefore, a current and readily accessible description of the LM in the feline and canine stifle would serve as an important veterinary anatomy learning resource. Our objective is to confirm the presence and anatomical characteristics of the LM in stifles of domestic canines and felines. We hypothesize that it is present as a normal stifle structure consisting of longitudinally arranged collagenous fibres interspersed with blood vessels and nerves. Our aim is to produce a detailed description of the LM to (1) serve as a foundation for advancing the understanding of stifle diseases; and to (2) facilitate student efforts to learn about stifle anatomy by differentiating this structure from other ligaments.

## 2 Methods

### 2.1 Animals

Animals for this project were either purchased commercially from Carolina Biological Supply (Burlington, NC) or were deceased patients presenting to the Matthew J. Ryan Veterinary Hospital of the University of Pennsylvania. Patient cadavers were either submitted for postmortem examination to the hospital's Autopsy Service or donated to its Educational Memorial Program.

The stifles of 62 hindlimbs from 32 cats were collected and used for this study. Breeds included 28 domestic shorthair, three domestic longhair, and one Siamese. Ages ranged from 2 months to 15½ years. Two stifles from two cats were excluded due to the removal of the distal femur and infrapatellar fat pad during the necropsy.

A total of 47 canine stifles were used from this study. Nine fixed stifles came from isolated canine hindlimbs, and the remaining 38 stifles were bilaterally collected from 19 fresh whole dogs. Breeds included six mixed, one Brittany Spaniel, one Goldendoodle, one Pekingese, one Great Dane, one Pit Bull, one Australian Shepherd, one German Shepherd, one Golden Retriever, one English Bulldog, one American Bulldog, one French Bulldog, one Cavalier King Charles Spaniel, and one Shih Tzu.

### 2.2 Gross Anatomical Dissection

Stifles were dissected by one of two approaches (described below), based on whether the stifles were previously opened. Width measurements of the LM were taken with a standard 15 cm ruler, and gross characteristics were recorded.

#### 2.2.1 Approach 1

For 42 feline and 24 canine stifles, a transverse incision was made at the cranial border of the tibial tuberosity. The patellar ligament was then transected at the tibial insertion and the synovium excised to allow its reflection. Stifle flexion allowed visualization of the infrapatellar fat pad and LM proximally/cranially.

#### 2.2.2 Approach 2

For the remaining 20 feline and 23 canine stifles, an anatomic pathology resident or rotating clinical student during diagnostic necropsy made one or more transverse incisions to “open” the stifle, transecting the patellar ligament and cruciate ligaments. The infrapatellar fat pad and LM were visible either proximally/cranially or distally.

### 2.3 Histology

Structures from fresh specimens less than 48 h postmortem were transected at their attachments, fixed in 10% formalin for 24–48 h, then transferred to 70% ethanol. Samples were submitted for embedding and staining with haematoxylin and eosin (H&E) to the University of Pennsylvania Perelman School of Medicine's Center for Molecular Studies in Digestive and Liver Diseases & Molecular Pathology and Imaging Core. Slides were reviewed via light microscopy by a board‐certified veterinary pathologist.

## 3 Results

### 3.1 Gross Anatomy

**FIGURE 2:**
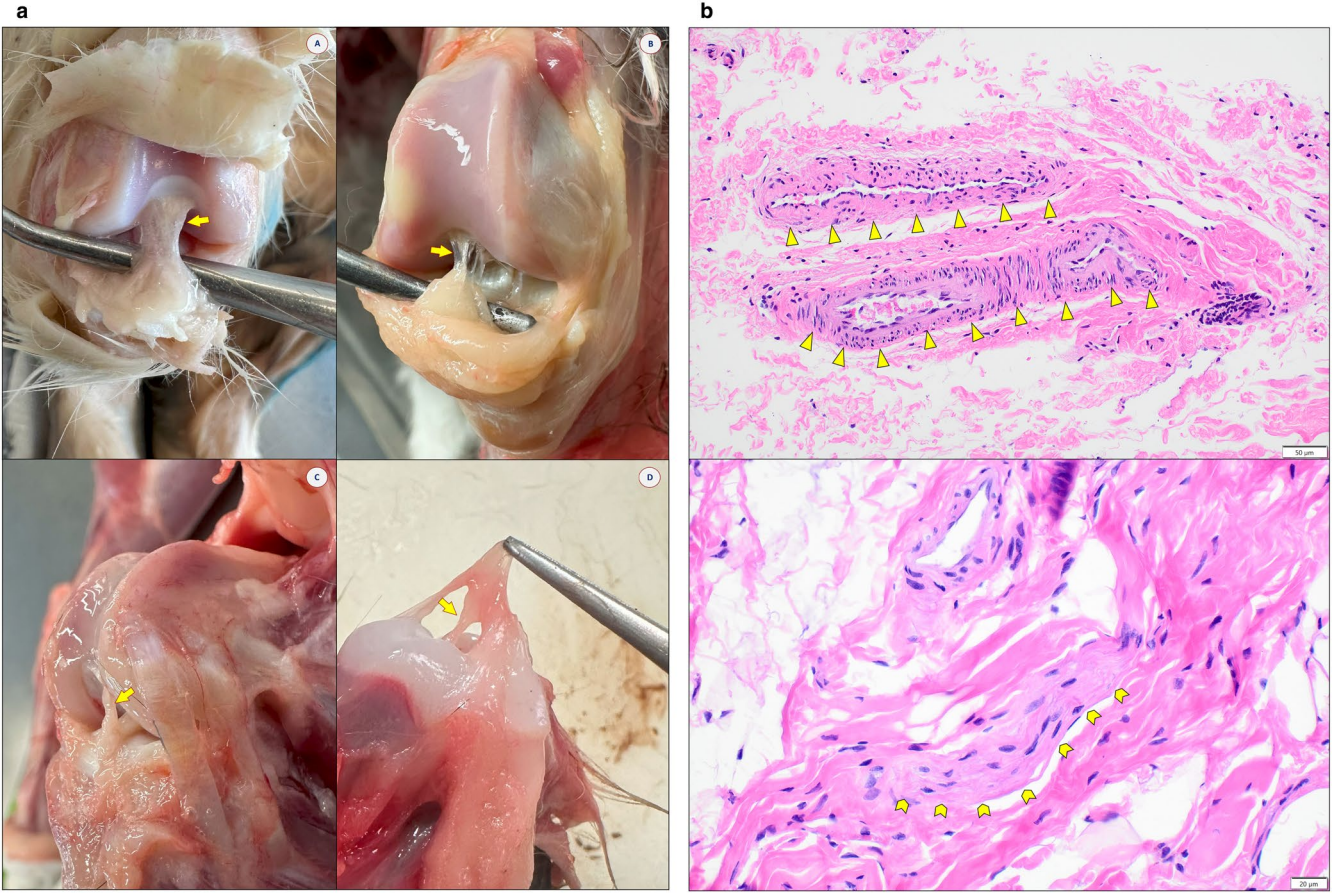
(a) Feline ligamentum mucosum (LM), anatomy. LM (arrow) observed in fixed (A), adult fresh (B, C), and juvenile fresh (D) hindlimb specimens. Note the individual variation in morphologies. (b) Feline ligamentum mucosum (LM), histology—H&E. Collagen present throughout sections. Top: Vascular tissue, 20× (arrowheads). Bottom: Neuronal tissue, 40× (chevron arrows).

**FIGURE 3:**
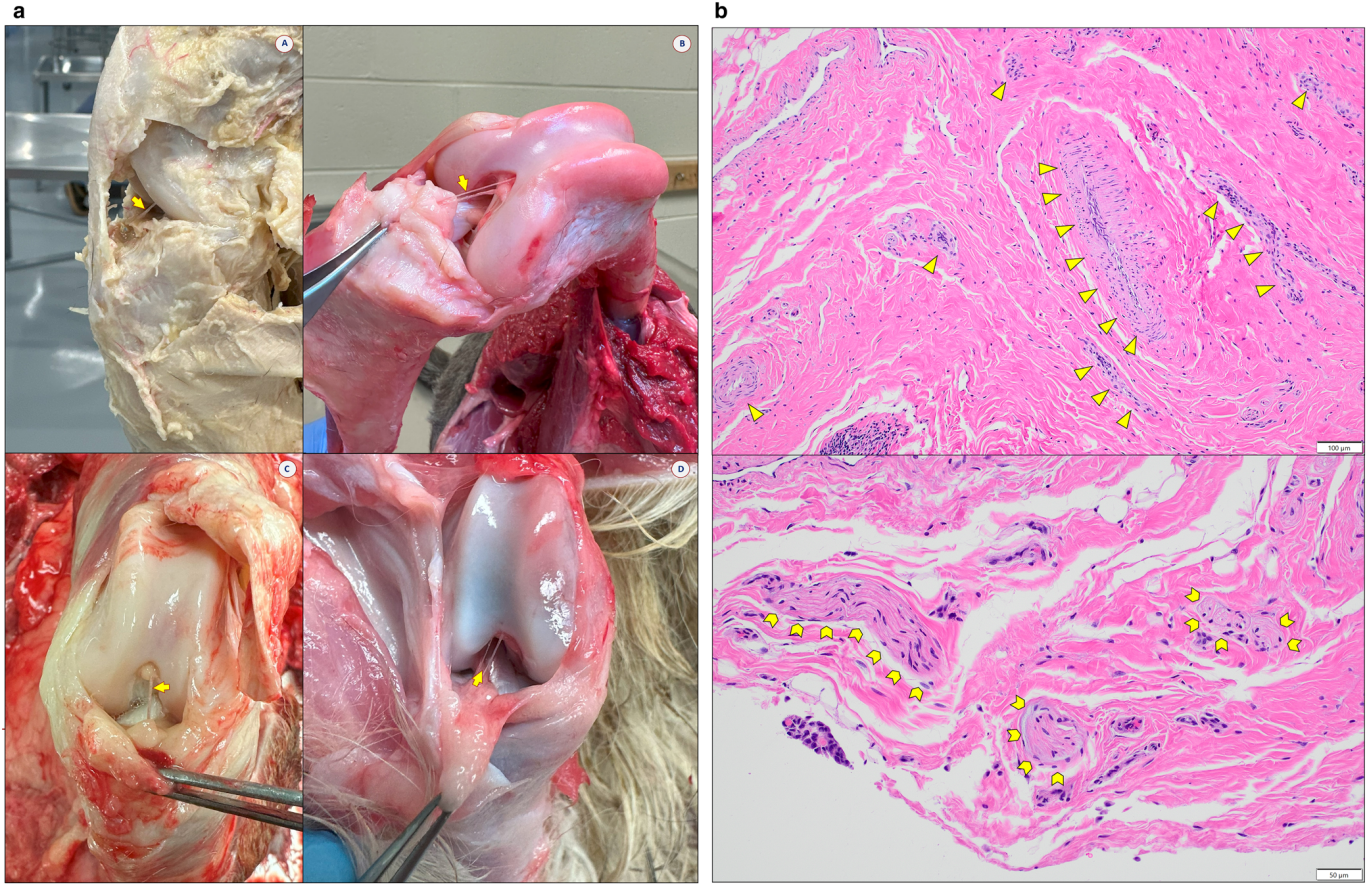
(a) Canine ligamentum mucosum (LM), anatomy. LM (arrow) observed in fixed (A) and adult fresh (B–D) hindlimb specimens. In (B, D), a vessel can be seen coursing parallel to the ligament. (b) *Canine ligamentum mucosum (LM), histology—H&E*. Collagen present throughout sections. Top: Vascular tissue, 10× (arrowheads). Bottom: Neuronal tissue, 20× (chevron arrows).

A white‐to‐pink, translucent, elastic, friable structure situated amongst variable amounts of fat and fascia was observed in 59 of 62 feline stifles and 39 of 47 canine stifles (Tables 1 and 2). The structure originated from the intercondylar notch of the femur, at the cranial border of the origin of the caudal cruciate ligament, and coursed into the central body of the infrapatellar fat pad (Figures 2a and 3a). In cats, its width typically measured 1–7 mm; in dogs, it typically measured ≤ 1 mm.

**TABLE 1:** Percentages of the presence of the LM in dissected feline stifles.

	Approach 1		Approach 2
	Fixed	Fresh	Fresh
**Number of Stifles**	24	18	20
**Presence of LM**	22 (92%)	18 (100%)	19 (95%)

**TABLE 2:** Percentages of the presence of the LM in dissected canine stifles.

	Approach 1		Approach 2
	Fixed	Fresh	Fresh
**Number of Stifles**	9	15	23
**Presence of LM**	9 (100%)	12 (80%)	18 (78%)

### 3.2 Histology

Microscopic examination revealed brightly eosinophilic collagen fibrils with vascular tissue present throughout (Figures 2b and 3b). Neurons were identified in some sections, and occasionally, sections included synovial tissue and synoviocytes in the periphery.

## 4 Discussion

**FIGURE 4:**
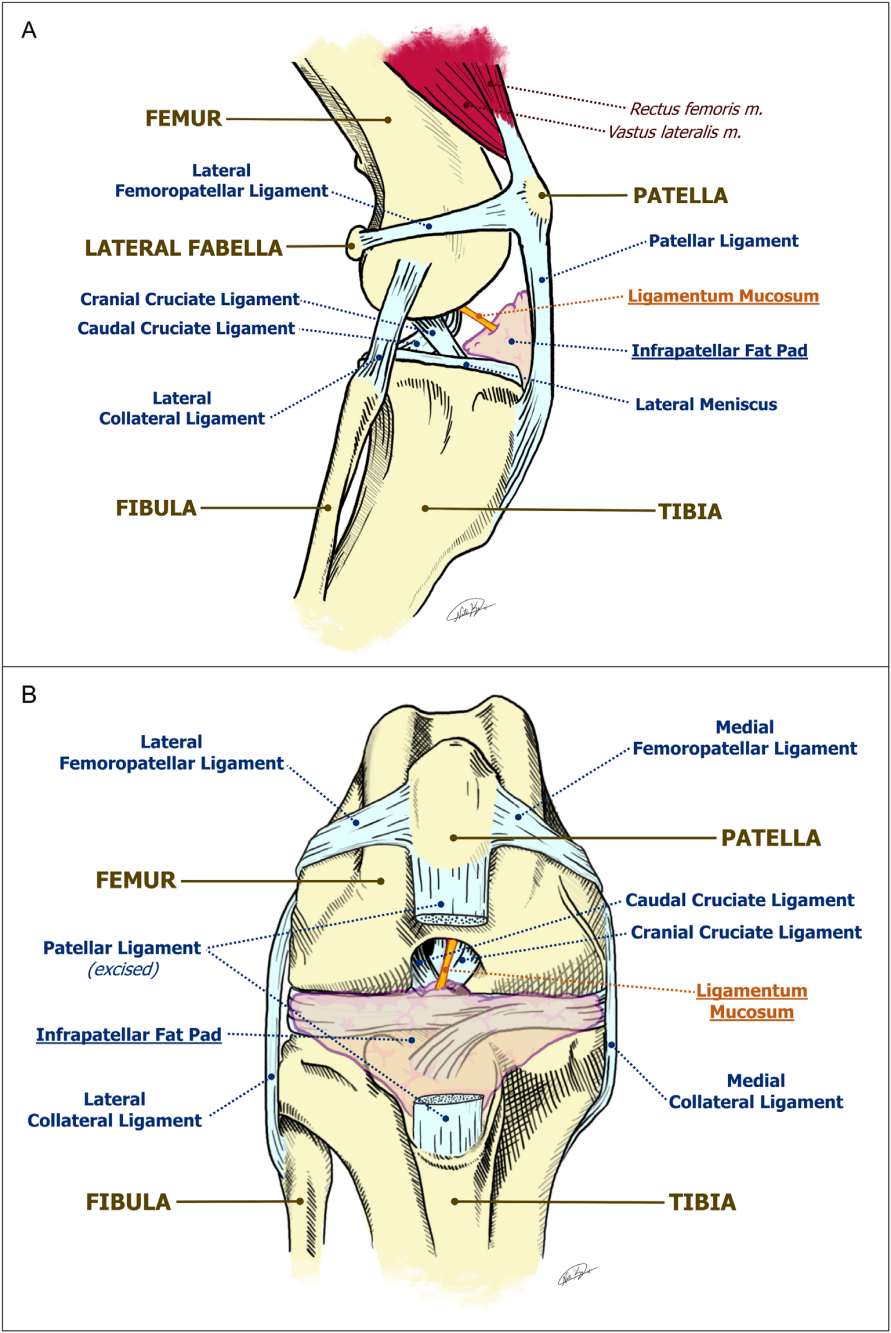
*Ligaments of the right canine stifle, ligamentum mucosum* (*LM*) *included*. (A) Cranial view. Infrapatellar fat pad obscures medial and lateral menisci. (B) Lateral view.

We describe a connective tissue structure anterior to the cruciate ligaments in 83.0% of domestic canine stifles and 95.2% of feline stifles examined. The predominance of dense collagen fibrils suggests its status as a ligament. In both cats and dogs, the ligament's attachment points and presence of both vascular and neuronal networks are consistent with descriptions of the LM in humans. Notably, said characteristics in the cat are consistent with a previous description in the dog (Sommer et al. 1999). These results constitute evidence that this structure is indeed the LM. Differences between the canine and feline LM remain unclear, though the canine LM was anecdotally thinner and associated with less loose connective tissue than that of the feline. In addition, anecdotally there also appeared to be more variation in the thickness of the feline LM compared to the canine LM (refer to Figures 2a and 3a). It is unclear if these observations in morphology are a reflection of anatomic differences across the larger population of felids and canids, nor is it clear what the physiological roles or reasons (if any) of/for such differences are. Nevertheless, these findings can aid in developing and improving educational resources for veterinary anatomy to include the LM (Figure 4), thereby reducing ambiguity and confusion that is sometimes apparent when learning the anatomy of the stifle.

Despite our inability to rule out false‐negative absences in stifles due to the friability of the ligament and the differences between the two dissection approaches, our findings describe a relatively high percentage of dogs and cats with the LM. Previous studies in human cadavers report a lower prevalence of about 65% in examined specimens (Nawfal et al. 2011).

Observed differences in gross appearance and width measurements may be due to individual variation, as observed in human knees, for which systems of classification have been proposed (Gonera, Kurtys, et al. 2023). Length measurements are not reported in this study due to breed differences in stifle dimensions and morphology; additionally, the stifle had to be flexed to visualize the LM, potentially stretching it beyond its length at relaxation. Although the embalmed animals in this study generally appeared to be healthy, young adult animals, we did not have any medical history for this subset. In addition, the client‐donated (fresh) cadavers typically arrived with more information about their background; however, some animals did have incomplete information. Therefore, we could not accurately ascertain any relationships between age or other factors. Other confounding factors may include postmortem condition and pathologic changes (if any) due to stifle disease.

To the authors' knowledge, this is the first report of the LM in the cat. Its presence in 1 out of 1 kitten hindlimb (Figure 2a,b) despite atrophy of the associated infrapatellar fat pad due to malnutrition suggests that it exists from birth or a very young age, consistent with embryological development of the LM as previously described (Casadei and Hermena 2023).

While the function and clinical relevance of the LM in humans have been postulated, it is unknown in animals. We recommend further research to characterize the LM in greater detail and investigate its function and clinical relevance. Regarding joint disease, in this study the articular surfaces of most joints appeared to have only mild or no changes upon gross inspection. Veterinary grading schemes for degenerative joint disease, such as the Canine OsteoArthritis Staging Tool (COAST) and the Feline Musculoskeletal Pain Index (FMPI), focus on antemortem clinical signs rather than histopathologic changes to joints (Cachon et al. 2018; Enomoto et al. 2022). Given that the current diagnostics for joint disease rely on evaluation for clinical lameness, these animals could not be graded on a conventional scale used to determine the severity of joint disease. With that, correlations of the LM with joint disease or other physiological factors are interesting questions that are outside of the scope of this study but surely warrant further investigation in a cohort of animals with more complete medical information. While controlling for health and disease states may be difficult due to differences in clinical presentations of degenerative joint disease, the scope of the ligament's veterinary clinical significance could be further discerned by confirmation of tissue types via additional histological and immunohistochemical stains; in situ visualization via imaging studies; and determination of load‐bearing properties via biomechanical testing, all of which have been performed in humans (Gonera, Kurtys, et al. 2023; Gonera, Wysiadecki, et al. 2023; Norris et al. 2018). Additionally, examining the stifles of other domestic and wild species to determine the presence of an LM could elucidate interspecies differences in stifle health and function.

## Funding

This work was supported by a grant from the National Institutes of Health (T35OD010919‐29), and a grant from Boehringer Ingelheim.

## Ethics Statement

Funding was obtained from the National Institutes of Health/Boehringer Ingelheim Summer Research Fellowship Program. The authors declare that there are no conflicts of interest. No artificial intelligence was used in either the conduction of this research or the writing/illustration of any part of this article.

## Conflicts of Interest

The authors declare no conflicts of interest.

## Data Availability

The data that support the findings of this study are available from the corresponding author upon reasonable request.
